# A Phenotypic-Driven Approach for the Diagnosis of WOREE Syndrome

**DOI:** 10.3389/fped.2022.847549

**Published:** 2022-04-29

**Authors:** Antonella Riva, Giulia Nobile, Thea Giacomini, Marzia Ognibene, Marcello Scala, Ganna Balagura, Francesca Madia, Andrea Accogli, Ferruccio Romano, Domenico Tortora, Mariasavina Severino, Paolo Scudieri, Simona Baldassari, Ilaria Musante, Paolo Uva, Vincenzo Salpietro, Annalaura Torella, Vincenzo Nigro, Valeria Capra, Lino Nobili, Pasquale Striano, Maria Margherita Mancardi, Federico Zara, Michele Iacomino

**Affiliations:** ^1^Unit of Medical Genetics, IRCCS Istituto Giannina Gaslini, Genoa, Italy; ^2^Department of Neuroscience, Rehabilitation, Ophthalmology, Genetics, Maternal and Child Health, University of Genoa, Genoa, Italy; ^3^Unit of Child Neuropsychiatry, Epilepsy Centre, Department of Medical and Surgical Neuroscience and Rehabilitation, IRCCS Istituto Giannina Gaslini, Genoa, Italy; ^4^Paediatric Neurology and Muscular Disease Unit, IRCCS Istituto Giannina Gaslini, Genoa, Italy; ^5^Department of Functional Genomics, Center for Neurogenomics and Cognitive Research (CNCR), Vrije Universiteit (VU) Amsterdam, Amsterdam, Netherlands; ^6^Division of Medical Genetics, Department of Specialized Medicine, Montreal Children’s Hospital, McGill University Health Centre (MUHC), Montreal, QC, Canada; ^7^Department of Human Genetics, McGill University, Montreal, QC, Canada; ^8^Neuroradiology Unit, IRCCS Istituto Giannina Gaslini, Genoa, Italy; ^9^Bioinformatica Clinica, IRCCS Istituto Giannina Gaslini, Genoa, Italy; ^10^Department of Precision Medicine, University of Campania “Luigi Vanvitelli”, Naples, Italy; ^11^Telethon Institute of Genetics and Medicine, Pozzuoli, Italy

**Keywords:** epilepsy, Exome sequencing (ES), WOREE syndrome, array-CGH analysis, *WWOX* gene

## Abstract

**Background:**

WOREE syndrome is a rare neurodevelopmental disorder featuring drug-resistant epilepsy and global developmental delay. The disease, caused by biallelic pathogenic variants in the *WWOX* gene, usually leads to severe disability or death within the first years of life. Clinicians have become more confident with the phenotypic picture of WOREE syndrome, allowing earlier clinical diagnosis. We report a boy with a peculiar clinic-radiological pattern supporting the diagnosis of WOREE syndrome.

**Methods:**

DNA was extracted from blood samples of the proband and his parents and subjected to Exome Sequencing (ES). Agarose gel electrophoresis, real-time quantitative PCR (Q-PCR), and array-CGH 180K were also performed.

**Results:**

ES detected a pathogenic stop variant (c.790C > T, p.Arg264*) in one allele of *WWOX* in the proband and his unaffected mother. A 180K array-CGH analysis revealed a 84,828-bp (g.chr16:78,360,803–78,445,630) deletion encompassing exon 6. The Q-PCR product showed that the proband and his father harbored the same deleted fragment, fusing exons 5 and 7 of *WWOX*.

**Conclusions:**

Genetic testing remains crucial in establishing the definitive diagnosis of WOREE syndrome and allows prenatal interventions/parental counseling. However, our findings suggest that targeted Next Generation Sequencing-based testing may occasionally show technical pitfalls, prompting further genetic investigation in selected cases with high clinical suspicion.

## Introduction

WOREE syndrome is a rare neurodevelopmental disorder caused by biallelic pathogenic variants in the *WWOX* gene (*MIM 605131). The disease classically features drug-resistant epilepsy (DRE), and global developmental delay, leading to severe disability and death within the first years of life (1, 2). Recently, increasing scientific interest has focused on dissecting the biological processes underlying this severe phenotype as *WWOX* encodes for a transcriptional regulator potentially involved either in tumor suppression or cell growth and differentiation (3, 4). Nevertheless, impaired neuronal migration and axonal conductance were showed to be highly causative for the phenotype in *Wwox*-null mice (1, 2).

Next-generation sequencing (NGS) technologies have widely increased our chance to diagnose this as well as other rare and neglected diseases. However, NGS is not always resolutive and some cases may remain unexplained. For example, either deletions or duplications could be lost during the analysis, preventing important diagnosis to be made (5). Hence, the relevance of recently developed technologies such as long-reads or of “ancillary” techniques which may be supportive. Here we report the case of a patient with a peculiar clinic-radiological pattern suggestive for a WOREE syndrome, forcing us to further investigate the underlying genetic etiology.

## Materials and Methods

### Blood Sample Collection, DNA Extraction and Exome Sequencing

Fresh blood sample was drawn from the affected individual and his unaffected parents. Genomic DNA was isolated from 1 ml of peripheral blood using QIAamp DNA Blood Midi kit (Qiagen). Genomic DNA was enriched with SureSelect Clinical research exome 54 Mb (Agilent Technologies). Exome sequencing (ES) runs were performed on the NovaSeq6000 platform (Illumina Inc., San Diego, CA, United States) with 150-base paired-end reads, using a standard Illumina pipeline as previously described (6). Mean sequencing depth of 100× and 95% of targeted regions covered at 20× depth was obtained. Paired-end reads were mapped to the reference human genome sequence (GRch37/hg19) using Burrows-Wheeler aligner (BWA) tool. Single-nucleotide polymorphisms (SNPs) and short deletion or insertion (indels) variants were called by CLC BioWorkbench software using the specific variant calling plugin and gnomAD databases. The variants were filtered for in-house variants controls. Variants were filtered according to genetic criteria for very rare variants (MAF ≤ 0.001), non-synonymous (NS), splice site (SS), insertions and deletions (indel) variants. Validation, parental origin of the resulting variants and family segregation studies were performed by Sanger sequencing. Copy number variants (CNVs) were analyzed by copy number variant detection tool (CLC Bio) to identify CNVs in target sequencing data.

### Agarose Gel Electrophoresis

Primer 3.0 software was used to design the PCR primers of exon 4F and exon 7R across the deletion fragment of exon 6 (WWOX_ex4F: CACCTACTTGGACCCAAGAC; WWOX_ex7R: AAAACATCCTGGAGGAGCTG). The RT-PCR derived from fibroblast extracts of patient and unaffected parents and age-matched neurotypical control was performed by TaKaRa LA Taq DNA Polymerase and the reaction was commenced with an initial 3-min denaturation step at 94°C, followed by 33 cycles of denaturation (94°C) for 30 s, annealing (60°C) for 40 s, and extension (72°C) for 70 s, and ended with a final extension step at 72°C for 7 min. The PCR single mutated allele products were purified and subjected to Sanger sequencing using an ABI3700 automated sequencer (PE Bio systems, Foster City, CA, United States). The Sanger sequencing results were examined and compared with the help of visual software such as Chromas Lite. Sanger sequencing was further used to identify the deletion.

### Real-Time Quantitative Polymerase Chain Reaction

The genomic DNA reference sequences of *WWOX* (hg19, NM_016373) was obtained from the University of California, Santa Cruz (UCSC) Genome browser database^1^. Primer 3.0 software was used to design the specific PCR primers. Real-time quantitative PCR (Q-PCR) reaction was carried out using the CFX96 Touch Real-Time PCR Detection System (Bio-Rad, Hercules, CA, United States). SYBR Green (Bio-Rad) was used as the fluorescent label. The total reaction system is a 15-μl final volume; each assay was performed in triplicate in *n* = 3 experiments.

### Array-CGH 180K Analysis

DNA was analyzed by Comparative Genome Hybridization, CGH-array, using the Human Genome CGH Microarray 4× 180K Kit, probe design 086332 (Agilent Technologies, Santa Clara, CA, United States), according to the manufacturer’s instructions. The Agilent platform is an oligonucleotide-based microarray with an average resolution of about 25 kb to detect copy number variations and loss of heterozygosity (LOH) of 4 Mb. Raw data were analyzed using the Genomic Workbench 7.0.40 software (Agilent). Altered chromosomal regions and breakpoints and LOH events were detected using ADM-1 (threshold 10) with 0.5 Mb window size to reduce false positives. For aberration detection, the diploid peak centralization algorithm and the legacy centralization algorithm were applied to set the most common ploidy to zero. This is needed to ensure that the zero point reflects the most common ploidy state. Chromosome positions were determined using GRCh37/hg19 (UCSC Genome Browser, see text footnote 1, release 7 July 2000).

## Results

### Clinical Features

**FIGURE 1:**
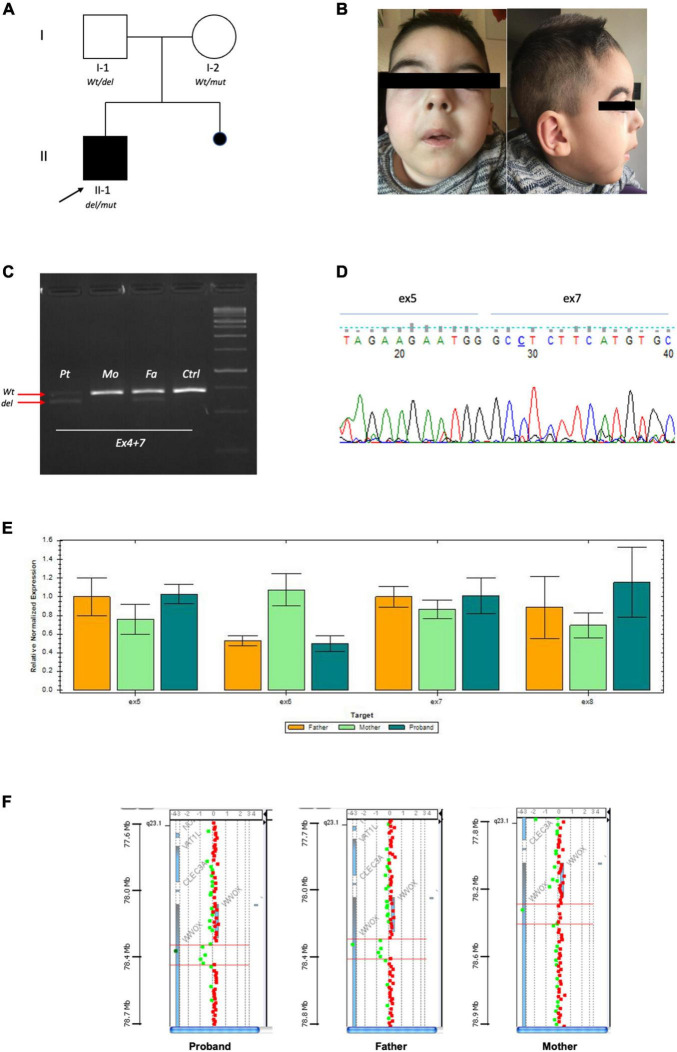
**(A)** Pedigree of family: Full square mutated affected case. Empty circle and square unaffected parents. **(B)** Portraits of patient II-1 at 4 years. **(C)** RT-PCR by fibroblasts showing that the proband and his father had the same product pattern. Arrows indicate the Wt product and deleted one. **(D)** Sanger of the deleted fragment showing fusion of exon 5 and 7. **(E)** Confirmative qPCR on DNA validating exon 6 deletions with loss of 50% product in the proband and carrier father. **(F)** High resolution array-CGH 180K highlighting genomic deletion of the proband and father.

**FIGURE 2:**
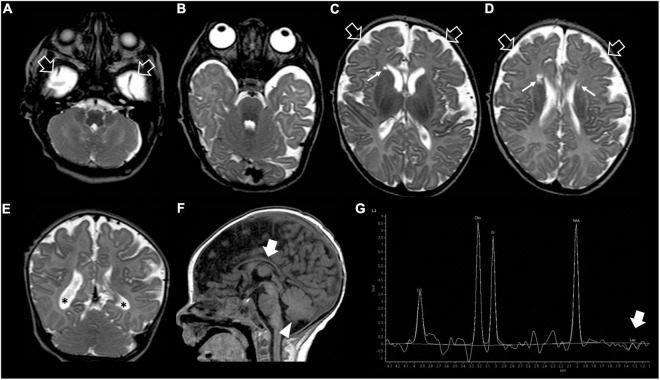
Brain MRI and MR spectroscopy at 4 months of age. **(A–D)** Axial and **(E)** Coronal T2-weighted images reveal delayed myelination, bilateral frontal periventricular cysts (arrows), enlarged subarachnoid spaces especially in the frontal-temporal regions (empty arrows), reduced white matter volume with mild lateral ventricle dilatation (asterisks). **(F)** Sagittal T1-weighted image shows hypoplasia of the corpus callosum (thick arrow) and inferior cerebellar vermis (arrowhead). **(G)** Brain MR spectroscopy performed at the level of the right basal ganglia demonstrates small lactate peaks (thick arrow).

**FIGURE 3:**
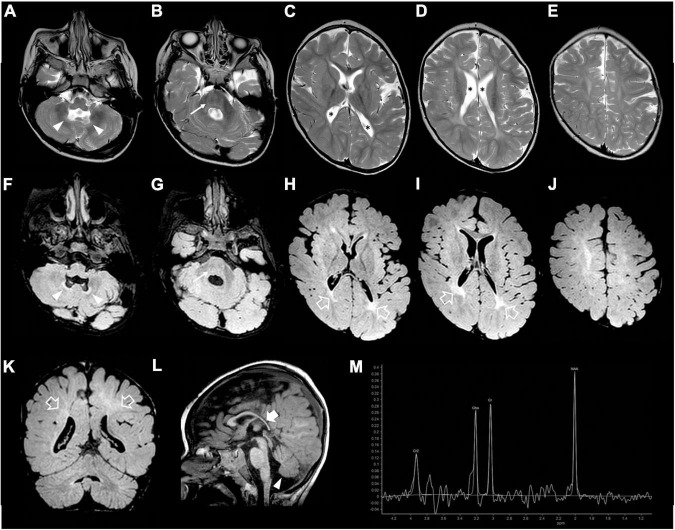
Brain MRI and MR spectroscopy were performed at 2 years and 4 months of age. **(A–E)** Axial T2-weighted, **(F–J)** axial FLAIR, and **(K)** coronal FLAIR images show mild evolution of the brain atrophy with white matter volume reduction and diffuse signal alterations, accentuated in the posterior periventricular regions (empty arrows). Note the squared lateral ventricles (asterisks). There are mild T2/FLAIR hyperintensities also at the level of the pons (arrows) and cerebellar dentate nuclei (arrowheads). **(L)** Sagittal T1-weighted image depicts marked thinning of the posterior sections of the corpus callosum (thick arrow), and inferior cerebellar vermis hypoplasia (arrowhead). **(M)** Brain MR spectroscopy performed at the level of the right basal ganglia shows a slightly reduced NAA peak and absence of lactate peaks.

Our patient was the first child of non-consanguineous parents coming from the same small Sicilian area, with unremarkable family history (Figure 1A). The boy was born at 36 weeks of gestation by C-section due to premature rupture of membranes. Birth weight was 3,100 g (90°centile), length 51 cm (97°centile), occipitofrontal circumference (OFC) 33 cm (50°centile), and Apgar score was 8 and 9 at 1 and 5 min. A few hours after birth, he presented hypoglycemia, respiratory distress associated to tonic-clonic seizures, and horizontal nystagmus. The electroencephalography (EEG) showed monomorphic delta activity with epileptiform discharges often accompanied by a sustained rhythmic jerking of the limbs. Brain magnetic resonance imaging (MRI) studies performed at the age of 7 days and 2 months showed frontal bilateral periventricular cysts, enlargement of cerebral subarachnoid spaces, especially in the frontal-temporal regions, and a small inferior vermis. He was evaluated for the first time at our hospital at the age of 4 months. Clinical and neurological evaluation revealed a lack of gaze fixation with horizontal nystagmus, axial hypotonia with spastic hypertonia of the limbs. Dysmorphic features included OFC 41 cm (25°centile), short neck, low anterior hairline, bushy eyebrows, long eyelashes, and broad nasal bridge (Figure 1B). EEG during wakefulness was characterized by slow monomorphic activity and multifocal independent spikes and spikes and waves. He presented multi-daily asymmetric tonic seizures (with eye and head deviation to the left, left arm hyperextension and right arm flexion) corresponding on EEG to a generalized high voltage slow wave with overlap of low amplitude fast activity and generalized voltage attenuation. Brain MRI at 4 months of age revealed delayed myelination, reduction of the white matter volume, and reabsorption of the periventricular cysts (Figure 2). Brain MR spectroscopy showed small lactate peaks. Over the years, the interictal epileptiform anomalies increased and the background activity became more disorganized. Several anti-seizure medications (ASM) were ineffective (i.e., valproate, vigabatrin, clonazepam, clobazam, levetiracetam, rufinamide, and CBD oil) or determined adverse events such as extreme drowsiness or increased secretions (i.e., phenobarbital, nitrazepam). Moreover, three cycles of ACTH had very poor results. The ketogenic diet did not significantly improve seizures and was suspended. Current ASMs include vigabatrin (70 mg/kg/day), clobazam (1 mg/kg/day) and clonazepam (0.9 mg/day). The multi-daily seizures progressed along with a severe movement disorder characterized by spastic tetraparesis and dystonia of limbs and trunk, worsening during periods of distress or discomfort. No developmental milestones were achieved. Brain MRI performed at 2 years and 4 months revealed mild progression of the brain atrophy and marked reduction with signal alterations of the periventricular white matter, especially in the parietal-occipital regions, thinned corpus callosum, and squared lateral ventricles. Mild signal alterations were also noted at the level of the pons and dentate nuclei (Figure 3). Brain MR spectroscopy demonstrated mild NAA reduction and the absence of lactate peaks (Figure 3). At 3 years, gastrostomy was placed due to failure to thrive and severe gastroesophageal reflux. At the last hospitalization, he showed profound intellectual disability, he was unable to follow objects and he never acquired sitting position. His severe spasticity and dystonia responded poorly to pharmacological treatment with baclofen. He developed scoliosis, and airway secretion accumulation required cough machine use. He presented bilateral ascending testis from the second month of life. The multi-daily seizures, up to 50 attacks per day, strongly disturbed his sleep. Specific tests for metabolic disorders (including neurotransmitters, folate, pterins, amino and organic acids, carnitine profile, plasma very long-chain fatty acids) were performed both on cerebrospinal fluid (CSF), blood and urine samples resulting within normal limits. Ophthalmological examination and auditory brain stem response did not show any pathological findings.

### Mutation Identification and Confirmation

Exome Sequencing detected the stop variant (NM_016373.4):c.790C > T (p.Arg264*) in one allele of *WWOX* in the proband and his unaffected mother. No other potentially pathogenic variants were identified in the exome. CNVs analysis was also unremarkable. Due to the strong clinical suspect, we performed additional analysis to reach genetic confirmation. The RT-PCR product (Figure 1C) showed the proband and his father had the same deleted exon 6 fragment (Figure 1C), fusing exon 5 and 7 of *WWOX* (Figures 1C,D). Q-PCR analysis showed both the proband and his father had the same deletion of exon 6 (Figure 1E), which by means of trio-based array-CGH 180K proved to be a 84,828-bp (g.chr16:78,360,803–78,445,630) deletion (Figure 1F).

## Discussion

The *WWOX* gene is located at 16q23.3-q24, the second most frequently chromosome fragile site in humans (FRA16D). It encodes a 46 KD, 414-amino acid protein that contains two WW domains at the NH2 terminus and a central short-chain dehydrogenase/reductase (SDR) domain (3). It is one of the most highly conserved proteins, and similar orthologs are present in Drosophila melanogaster, human, and mouse with 93.9% of proteins identity sharing. It is not unexpected that genes included in fragile chromosomal sites are implicated in carcinogenesis. This concept also applies to *WWOX*, which was initially widely described as a tumor suppressor gene involved in multiple tumor types (7).

In 2009, Suzuki et al. highlighted that *WWOX* pathogenic variants are involved in mice’s epileptogenesis (8). The growing enrichment of the scientific literature about the *WWOX* pathogenic variant has delineated a broad impairment in neurological disorders such as Alzheimer’s disease, multiple sclerosis, autism spectrum disorders, spinocerebellar ataxia, and epileptic encephalopathy (9). *WWOX* knockout animal models have demonstrated reduced concentrations of GABA synthesis accompanied by hippocampal gabaergic interneurons and Purkinje cell loss. Moreover, research studies on human neural progenitor cells (hNPCs) have shown a compromised cytoskeleton organization, thus likely contributing to the disruption of neuronal migration in the developing cerebral cortex, hippocampus, and cerebellum (10).

There are two main neuropathological phenotypes correlated with *WWOX* bi-allelic pathogenic variants: SCAR12 spinocerebellar ataxia-12 (SCAR12 syndrome) and *WWOX*-related epileptic encephalopathy (WOREE) (9, 11). *WWOX* missense mutations lead to hypomorphic alleles related to SCAR12 syndrome, an early-childhood onset cerebellar ataxia associated with non-progressive microcephaly, generalized tonic-clonic epilepsy, and developmental delay (12, 13). On the other hand, a complete lack of *WWOX* expression correlates with WOREE. In 2015, for the first time, Mignot et al. defined WOREE as severe, precocious, and refractory epileptic encephalopathy corresponding to multi-daily and polymorphic type of seizures with progressive microcephaly, severe global developmental delay, ophthalmological involvement, and premature death (14). This second phenotype is more severe than SCAR12 syndrome, especially concerning the severity of epilepsies. Previous studies (11, 15), describe 54 patients with WOREE syndrome associated with biallelic *WWOX* pathogenic variants. The most-reported clinical features are acquired microcephaly, severe and polymorphic seizures, and neurodevelopmental delay. Although genotype-phenotype correlation is still critical in genetic diagnosis, only a minority (41%) of the previous reports described the clinical phenotypes in detail (Supplementary Table 1).

In the present case, the main characteristics that lead to the diagnosis of WOREE were the dysmorphic features, the severity of epilepsy, and mostly the neuroradiological pattern. In particular, the patient had round hypotonic face, short neck, low anterior hairline, bushy eyebrows, long eyelashes, broad nasal bridge, and acquired microcephaly (11, 15). Nevertheless, dysmorphic features are not a constant feature of the whole WOREE phenotype since they may be absent in ∼52% of the patients (Supplementary Table 1) (11, 14). The average epilepsy onset in WOREE is 2–3 months (range 1 day to 7 months) (10, 15). Interestingly, our patient had the earliest epilepsy onset, presenting on the first day of life, and his multi-daily tonic seizures never responded to any pharmacological treatment. A recent review did not highlight a clear correlation between the extension of *WWOX* mutation and the onset or severity of epilepsy (9). Neurodevelopment is always severely impaired in WOREE, sometimes even before epilepsy onset (11, 16). Our patient showed spontaneous mobility, with a dystonic component occasionally observed in WOREE cases (11).

Regarding the neuroimaging features of WOREE, structural brain abnormalities including corpus callosum hypoplasia, progressive cerebral atrophy, cerebellar vermis hypoplasia, and white matter hyperintensity representing delayed myelination have been described in most cases. Similarly, our patient showed mild progressive brain atrophy, delayed myelination, and inferior cerebellar vermis hypoplasia. Interestingly, in the later phase of the disease, we noticed a peculiar leukoencephalopathy pattern, characterized by marked volume reduction and signal alterations of the periventricular white matter, especially in the posterior regions, with squared lateral ventricles and thinned corpus callosum, like in the prematurity-related periventricular leukomalacia. At the moment, there are no data on the evolution of neuroimaging findings in the late phases of WOREE, and more studies are needed to verify if this periventricular leukomalacia-like pattern is a typical feature of the disease.

Nowadays, clinicians have become much more confident with the phenotypic picture of WOREE syndrome, allowing early clinical diagnosis to be made. Yet, genetics is pivotal to making the definitive diagnosis and for parental counseling. However, targeted NGS strategies may show some pitfalls as small CNVs (encompassing one exon as in our case) may get lost, but if the clinical suspicion is strong it is worth performing further genetic investigations.

## Data Availability Statement

The datasets presented in this study can be found in online repositories. The names of the repository/repositories and accession number(s) can be found in the article/ Supplementary Material.

## Ethics Statement

Written informed consent was obtained from the minor(s)’ legal guardian for the publication of any potentially identifiable images or data included in this article.

## Author Contributions

AR, GN, and MI: conceptualization, writing – original draft, writing, review, and editing lead. TG: writing – original draft. MO, MSc, GB, FM, AA, FR, DT, MSe, PSc, SB, IM, PU, VS, AT, VN, VC, LN, PSt, and MM: writing, review, and editing support. FZ and MI: conceptualization, funding acquisition, supervision, writing review, and editing. All authors contributed to manuscript revision, read, and approved the submitted version.

## Conflict of Interest

AR has received honoraria from Kolfarma srl and Proveca Pharma Ltd. PSt has served on a scientific advisory board for the Italian Agency of the Drug (AIFA); has received honoraria from GW pharma, Kolfarma srl, Proveca Pharma Ltd., and Eisai Inc.; and has received research support from the Italian Ministry of Health and Fondazione San Paolo. The remaining authors declare that the research was conducted in the absence of any commercial or financial relationships that could be construed as a potential conflict of interest.

## Publisher’s Note

All claims expressed in this article are solely those of the authors and do not necessarily represent those of their affiliated organizations, or those of the publisher, the editors and the reviewers. Any product that may be evaluated in this article, or claim that may be made by its manufacturer, is not guaranteed or endorsed by the publisher.

## Footnotes

1
https://genome.ucsc.edu/
