# Supplementary material for: A Phenotypic-Driven Approach for the Diagnosis of WOREE Syndrome
Source: Front Pediatr. 2022 Apr 29;10:847549. doi: 10.3389/fped.2022.847549 (PMC9100683; doi:10.3389/fped.2022.847549)
Supplement: Supplementary file 1 [file Table_1.docx]

**Supplementary Table 1**. Phenotypic picture of our case compared to those from the literature.

|  | **This study** | **Abdel-Salam G. et al. 2014** | **Ben-Salem S.**  **et al. 2014** | **Mignot C.**  **et al. 2015** | **Tabarki B.**  **et al. 2015** | **Valduga M.**  **et al. 2015** | **Elsaadany L.**  **et al. 2016** | **Tarta-Arsene O.**  **et al. 2017** | **Serin H.M.**  **et al. 2018** | **Johannsen J.**  **et al. 2018** |
| --- | --- | --- | --- | --- | --- | --- | --- | --- | --- | --- |
| **N° of pts** | 1 | 1 | 1 | 5 | 5 | 2* | 2 | 1 | 1 | 2 |
| **Gender** | M | F | M | 4F/1M | 3F/2M | F/M | F 2/2 | M | F | F 2/2 |
| **Consanguinity** | - | + | + | - | 5/5 | 2/2 | 2/2 | - | + | 2/2 |
| **Ethnicity** | Italian | Egyptian | Emiratis | NR | Emiratis | Turkish | NR | NR | NR | Arabs |
| **Acquired mpch.** | + | + | + | 2/5 | - | 2/2 | -/- | - | + | 2/2 |
| **Dysmorphism** | + | + | - | - | NR | NR/NA* | -/- | - | NR | NR |
| **DD** | + | + | + | + | 5/5 | +/NA* | 2/2 | + | + | 2/2 |
| **Axial hypotonia** | + | NR | + | 5/5 | - | -/NA* | -/- | + | + | 2/2 |
| **Spasticity /Pyramidal signs** | + | +/+ | + | 2/5 | 5/5 | +/NA* | 2/2 | - | + | 2/2 |
| **Movement disorders** | + | - | - | - | - | -/NA* | -/- | - | - | - |
| **Ophthalmological involvement** | + | + | + | 3/5 | 2/5 | +/NA* | 2/2 | + | + | 2/2 |
| **Epilepsy (onset)** | 1d | 2m | 2w | Mean age of 3m  (range from 2m to 5m) | Mean age of 2m  (range from 2m to 3m) | 3m/probably prenatal* | 7w/7w | 4w | 2m | 2m/2m |

|  | **This study** | **Abdel-Salam G. et al. 2014** | **Ben-Salem S.**  **et al. 2014** | **Mignot C.**  **et al. 2015** | **Tabarki B.**  **et al. 2015** | **Valduga M.**  **et al. 2015** | **Elsaadany L.**  **et al. 2016** | **Tarta-Arsene O.**  **et al. 2017** | **Serin H.M.**  **et al. 2018** | **Johannsen J.**  **et al. 2018** |
| --- | --- | --- | --- | --- | --- | --- | --- | --- | --- | --- |
| **Sz types** | Asymmetric tonic sz | Focal and generalized TC, myoclonic sz | Asymmetric tonic sz | Focal tonic, generalized and multifocal sz, IS | IS 3/5,  secondarily generalized sz 3/5,  LGS 2/5 | Myoclonic sz and IS | Multifocal myoclonic jerks and asymmetric tonic sz | Secondarily generalized seizures and IS | IS | Tonic and myoclonic sz |
| **EEG**  **pattern** | Slow monomorphic activity, multifocal independent SW | Generalized epileptic abnormalities with frequent bursts of sharp and slow waves | Hypsarrhythmia | SBA 5/5,  Posterior SW 3/5,  Anterior SW 1/5, Multifocal SW 1/5,  close to hypsarrythmia 1/5 | NR | Hypsarrhythmia | SBA and multifocal spikes 1/2, continuous generalized slowing and multifocal spikes 1/2 | Hypsarrhythmia | Hypsarrhythmia | Hypsarrhythmia |
| **Response**  **to AED** | DR | Partial | Complete | Partial 5/5 | DR 5/5 | Partial | DR/Partial | DR | Partial | DR |
| **Brain MRI** | Brain atrophy, thin CC, inferior cerebellar vermis hypoplasia, periventricular leukomalacia-like pattern | Brain atrophy | Cerebral atrophy, right frontoparietal region PMG | Brain atrophy 1/5,  thin CC 1/5,  myelination  delay 1/5,  normal MRI 2/5 | Brain atrophy 2/5,  thin CC 2/5,  Wallerian degeneration 2/5, MTN lesions 1/5 | Brain atrophy 2/2, PMG 1/2 | Progressive demyelination, brain atrophy 2/2 | Brain atrophy, thin CC and Wallerian degeneration | Temporal lobes atrophy | Brain atrophy 2/2, thin CC 2/2, myelination delay 1/2 |
| **Feeding difficulties** | + | - | - | NR | NR | -/NA* | +/+ | NR | NR | +/+ |
| **Scoliosis/**  K**yphosis** | S | Too young | - | Too young or NR | Too young or NR | S/NA* | -/S | NR | NR | NR/NR |
| **Other** | Cryptorchidism, respiratory problems | - | Bilateral inguinal hernias | NR | NR | P1:Cardiomyopathy | Other | Cryptorchidism, respiratory problems | - | Bilateral inguinal hernias |

|  | **This study** | **Abdel-Salam G. et al. 2014** | **Ben-Salem S.**  **et al. 2014** | **Mignot C.**  **et al. 2015** | **Tabarki B.**  **et al. 2015** | **Valduga M.**  **et al. 2015** | **Elsaadany L.**  **et al. 2016** | **Tarta-Arsene O.**  **et al. 2017** | **Serin H.M**  **et al. 2018** | **Johannsen J.**  **et al. 2018** |
| --- | --- | --- | --- | --- | --- | --- | --- | --- | --- | --- |
| **Premature**  **death** | No | 16m | No | 2/5 (16m;38m) | 5/5 (<3yrs) | <3yrs/39w* | No/No | 3yrs | No | No |
| **Mutations** | CH:  Stop gained exon and del. exon 6 | HMZ stop gained exon 2 | HMZ del.  exon 5 | P1:CH, several exons del.  P2: CH, exon 6 del. and nonsense  exon 8  P3: CH, frameshift exon 1+missense exon 2  P4: NR  P5: CH, complete del.+ nonsense  exon 8 | 5/5 HMZ del. | 2/2 HMZ del.  exon 1-6 | HMZ stop gained exon 2 | CH: intron 2 + frameshift exon 8 | HMZ missense exon 7 | HMZ missense exon 3 |

|  | **Davids M.**  **et al. 2018** | **Shaukat Q.**  **et al. 2018** | **Yang C.**  **et al. 2019** | **Piard J.**  **et al. 2019** | **Weisz-Hubshman M.**  **et al. 2019** | **He J.**  **et al. 2019** | **Su T.**  **et al. 2019** | **Iacomino M.**  **et al. 2020** |
| --- | --- | --- | --- | --- | --- | --- | --- | --- |
| **N° of pts** | 1 | 2 | 1 | 20 | 6 | 1 | 1 | 2* |
| **Gender** | F | M 2/2 | M | 12F/8M | 4F/2M | M | F | M/NR* |
| **Consanguinity** | - | 2/2 | NR | 5/20 | 2/6 | NR | No | No/No |
| **Ethnicity** | Caucasian | NR | Chinese | European 10/20  Asia 4/20  Africa 4/20  Unknow 2/20 | Yemenite Jews | Chinese | Chinese | Italian |
| **Acquired**  **mpch** | + | 2/2 | - | 4/20 | 6/6 | - | + | +/NA* |
| **Dysmorphism** | + | NR | - | 12/20 | 3/6 | NR | NR | +/NA* |
| **DD** | + | 2/2 | + | 20/20 | 6/6 | + | + | +/NA* |
| **Axial hypotonia** | + | 2/2 | + | 15/20 | 4/6 | + | + | +/NA* |
| **Spasticity /Pyramidal signs** | + | 2/2 | - | 15/18 | 4/6 | - | + | +/NA* |
| **Movement disorders** | - | -/- | - | 6/18 | - | - | - | +/NA* |
| **Ophthalmological involvement** | + | 2/2 | - | 18/20 | 4/6 | + | + | +/NA* |
| **Epilepsy (onset)** | 5w | 7m/5w | 3w | Mean age of 1.6 m  (range from 1d to 7 m) | Mean age of 5w  (range from 2w to 3 m) | 1m | 55d | 45d/NA* |
| **Sz types** | TC and myoclonic sz | IS and tonic sz 1/2,  IS, lip smacking and arm jerking 1/2 | Asymmetric tonic or clonic sz | Secondarily generalized sz 9/20, focal clonic or tonic sz 7/20, generalized sz 5/20,  IS 8/20, LGS 2/20 | IS 1/6,  TC sz 1/6,  rhythmic movements 1/6,  focal tonic 3/6,  myoclonic 1/6 | Myoclonic sz, IS | Focal myoclonic sz, IS | TC, tonic, and myoclonic sz/NA* |
| **EEG** | SBA and SW | Hypsarrhythmia | Low-amplitude fast waves in the left central region | SBA 8/18, hypsarrhythmia 5/18, posterior epileptic discharge 4/18, multifocal epileptic discharges 11/18 | Multifocal epileptic discharges 1/6,  focal discharge 4/6,  hypsarrhythmia 1/6, NR 1/6 | SW on posterior region, slow posterior activity | Hypsarrhythmia | Burst suppression-like pattern /NA* |
| **Response to ASMs** | Partial | Partial | Partial | DR 19/20 | DR 4/6  Partial 2/6 | DR | Complete | DR/NA* |

|  | **Davids M.**  **et al. 2018** | **Shaukat Q.**  **et al. 2018** | **Yang C.**  **et al. 2019** | **Piard J.**  **et al. 2019** | **Weisz-Hubshman M.**  **et al. 2019** | **He J.**  **et al. 2019** | **Su T.**  **et al. 2019** | **Iacomino M.**  **et al. 2020** |
| --- | --- | --- | --- | --- | --- | --- | --- | --- |
| **Brain MRI** | Cortical atrophy, thin CC and cerebellar vermis, enlargement of ventricles, and prominent cisterns, globus pallidus with iron deposits | Temporal lobes atrophy,  thin CC, periventricular connatal cysts 1/2.  Temporal lobes atrophy,  bilateral parietal PMG, dysplastic thin CC 1/2 | Normal | Thin CC 15/20, progressive cerebral atrophy 11/20, delayed myelination 1/20 | Thin CC, moderate brain atrophy and delayed myelination 2/6, small vermis 1/6, isolated brain atrophy 1/6, isolated thin CC 2/6, NA 1/6 | Thin CC, cerebral atrophy | Cerebral atrophy | Cerebral atrophy, thin CC and cerebellar vermis 1/2  Mild cerebellar vermis hypoplasia 1/2** |
| **Feeding difficulties** | + | -/- | NR | 13 +/ 1 NA | P3 + | NR | NR | +/NA* |
| **Scoliosis/**K**yphosis** | S | NR/NR | NR | 9 S/ 4 K /2 S+K | Too young or NR | Too young | Too young | -/NA* |
| **Other** | Tracheostomy,  laryngomalacia,  WPW syndrome | P2: Tracheostomy | NR | 8 respiratory problem 1 cryptorchidism | P3: Tracheostomy  P6: MPS, ASD,  hydro-nephrosis | NR | NR | Cryptorchidism/NA* |
| **Premature death** | No | No | No | Mean age of 40m 8/20 (range from 6m to 8yrs) | 1/6 (9m) | 1yrs | 5m | No/NA* |
| **Mutations** | HMZ del. exon 6 | P1: HMZ del. exons 3 to 4  HMZ splice site intron 6 | CH missense  and splice site exon 9 | HMZ dup.1/20  HMZ del. 2/20  HMZ missense 1/20  HMZ stop gained 2/20  CH del. 2/20  CH missense + del.4/20  CH Missense and frameshift 1/20  CH Missense 3/20  CH stop gained + frameshift 1/20  CH splice site + dup.1/20  CH del.+ frameshiftit 1/20  CH frameshift 1/20 | P1, P3: HMZ  P6: CH | CH missense | CH del. + dup. | HMZ stop gained 2/2 |

**Legend:** ASMs-Anti-seizure medications, ASD-mild atrial-septal defect, CC-Corpus Callosum, CH-Compound Heterozygous, d-days, del.-deletion, DR-Drug Resistant, dup.-duplication, EEG- Electroencephalogram, F-female, HMZ- Homozygous, IS-Infantile Spasm, LGS-Lennox- Gastaut Syndrome, K-Kyphosis, M-male, m-months, Mpch-Microcephaly, MPS-mild pulmonic stenosis, MRI-Magnetic Resonance Imaging, MTN-Medial Thalamic Nuclei, NA-data Not Aviable, NR-data Not Reported, P-patient, PMG-Polymicrogyria, Psychom. Delay-Psychomotor Delay, SBA-slow background activity, S-Scoliosis, SW-spikes and waves, TC-Tonic-Clonic, w-weeks, yrs-years, WPW- Wolff–Parkinson–White, *-fetus terminated, **-prenatal imaging.

**Supplementary References**

Abdel-Salam G, Thoenes M, Afifi HH, Körber F, Swan D, Bolz HJ. The supposed tumor suppressor gene WWOX is mutated in an early lethal microcephaly syndrome with epilepsy, growth retardation and retinal degeneration. *Orphanet J Rare Dis*. 2014 Jan 23;9:12.

Ben-Salem S, Al-Shamsi AM, John A, Ali BR, Al-Gazali L. A novel whole exon deletion in WWOX gene causes early epilepsy, intellectual disability and optic atrophy. *J Mol Neurosci*. 2015 May;56(1):17-23.

Tabarki B, AlHashem A, AlShahwan S, Alkuraya FS, Gedela S, Zuccoli G. Severe CNS involvement in WWOX mutations: Description of five new cases. *Am J Med Genet A.* 2015 Dec;167A(12):3209-13.

Valduga M, Philippe C, Lambert L, Bach-Segura P, Schmitt E, Masutti JP, et al. WWOX and severe autosomal recessive epileptic encephalopathy: first case in the prenatal period. J Hum Genet. 2015 May;60(5):267-71.

Elsaadany L, El-Said M, Ali R, Kamel H, Ben-Omran T. W44X mutation in the WWOX gene causes intractable seizures and developmental delay: a case report. *BMC Med Genet*. 2016 Aug 5;17(1):53.

Tarta-Arsene O, Barca D, Craiu D, Iliescu C. Practical clues for diagnosing WWOX encephalopathy. *Epileptic Disord.* 2017 Sep 1;19(3):357-361.

Serin HM, Simsek E, Isik E, Gokben S. WWOX-associated encephalopathies: identification of the phenotypic spectrum and the resulting genotype-phenotype correlation. *Neurol Sci*. 2018 Nov;39(11):1977-1980.

Johannsen J, Kortüm F, Rosenberger G, Bokelmann K, Schirmer MA, Denecke J, et al. A novel missense variant in the SDR domain of the WWOX gene leads to complete loss of WWOX protein with early-onset epileptic encephalopathy and severe developmental delay. *Neurogenetics*. 2018 Aug;19(3):151-156.

Davids M, Markello T, Wolfe LA, Chepa-Lotrea X, Tifft CJ, Gahl WA, et al. Early infantile-onset epileptic encephalopathy 28 due to a homozygous microdeletion involving the WWOX gene in a region of uniparental disomy. *Hum Mutat*. 2019 Jan;40(1):42-47.

Yang C, Zhang Y, Song Z, Yi Z, Li F. Novel compound heterozygous mutations in the WWOX gene cause early infantile epileptic encephalopathy. *Int J Dev Neurosci*. 2019 Dec;79:45-48.

He J, Zhou W, Shi J, Zhang B, Wang H. A Chinese patient with epilepsy and WWOX compound heterozygous mutations. *Epileptic Disord.* 2020 Feb 1;22(1):120-124.

Su T, Yan Y, Xu S, Zhang K, Xu S. Early onset epileptic encephalopathy caused by novel compound heterozygous mutation of WWOX gene. *Int J Dev Neurosci.* 2020 Apr;80(2):157-161.
